# Electrical circuit model of ITO/AZO/Ge photodetector

**DOI:** 10.1016/j.dib.2017.07.031

**Published:** 2017-07-14

**Authors:** Malkeshkumar Patel, Joondong Kim

**Affiliations:** Photoelectric and Energy Device Applications Lab (PEDAL) and Department of Electrical Engineering, Incheon National University, 119 Academy Rd. Yeonsu, Incheon 406772, Republic of Korea

**Keywords:** ITO/AZO/Ge photodetector, R-C circuit model, Heterojunction, Impedance spectroscopy

## Abstract

In this data article, ITO/AZO/Ge photodetector was investigated for electrical circuit model. Due to the double (ITO and AZO) transparent metal-oxide films (DOI:10.1016/j.mssp.2016.03.007) (Yun et al., 2016) [1], the Ge heterojunction device has a better interface quality due to the AZO layer with a low electrical resistance due to the ITO layer (Yun et al., 2015) [2]. The electrical and interfacial benefitted ITO/AZO/Ge heterojunction shows the quality Schottky junction. In order to investigate the device, the ITO/AZO/Ge heterojunction was analyzed by R–C circuit model using the impedance spectroscopy.

## Specifications Table

**Table:** 

Subject area	*Physics, Electrical Engineering*
More specific subject area	*Photodetector*
Type of data	*Figures, Table*
How data was acquired	*Potentiostat/Galvanostat (ZIVE SP1, WonA Tech, Korea)*
Data format	*Analyzed*
Experimental factors	*Impedance spectroscopy: Frequency range 1 Hz to 20 kHzBias range → −0.5 V to 0.5 V in step of 0.1 V*
Experimental features	*Double transparent metal-oxide films (ITO/AZO)*
Data source location	*Incheon National University, Incheon 22,012, Korea*
Data accessibility	*The data are with this article*
*The Potentiostat/Galvanostat was calibrated with a standard static and dynamic circuit before the impedance and Mott-Schottky measurement*.	

## Value of the data

• R-C electric circuit was adopted to investigate the metal oxide/Ge photodetector.
• Double transparent metal-oxide films were adopted to enhance the electrical and interfacial properties.
• The bias dependent impedance spectra including the Nyquist and Bode plots demonstrated the functional modulation of the space charge region of ITO/AZO/Ge photodetector.

## 1 Data

**Fig. 1:**
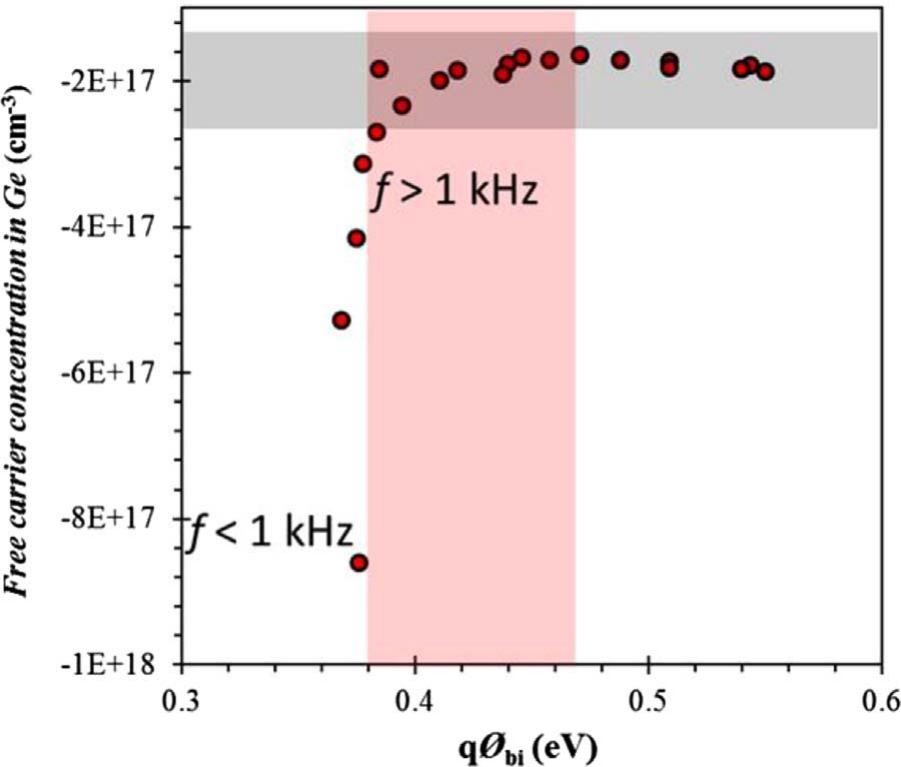
Profile of the free carrier concentrations of the ITO/AZO/Ge device as a function of built in potential.

**Fig. 2:**
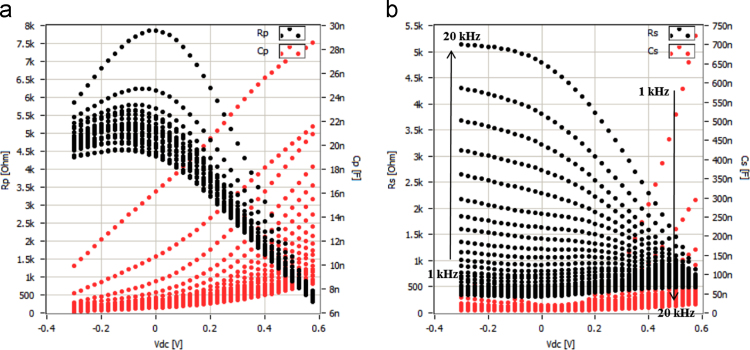
(a) Parallel resistance (*R*_p_) and parallel capacitance (*C*_p_) as a function of bias operation, and (b) series resistance (*R*_S_) and series capacitance (*C*_S_) as a function of bias operation.

**Fig. 3:**
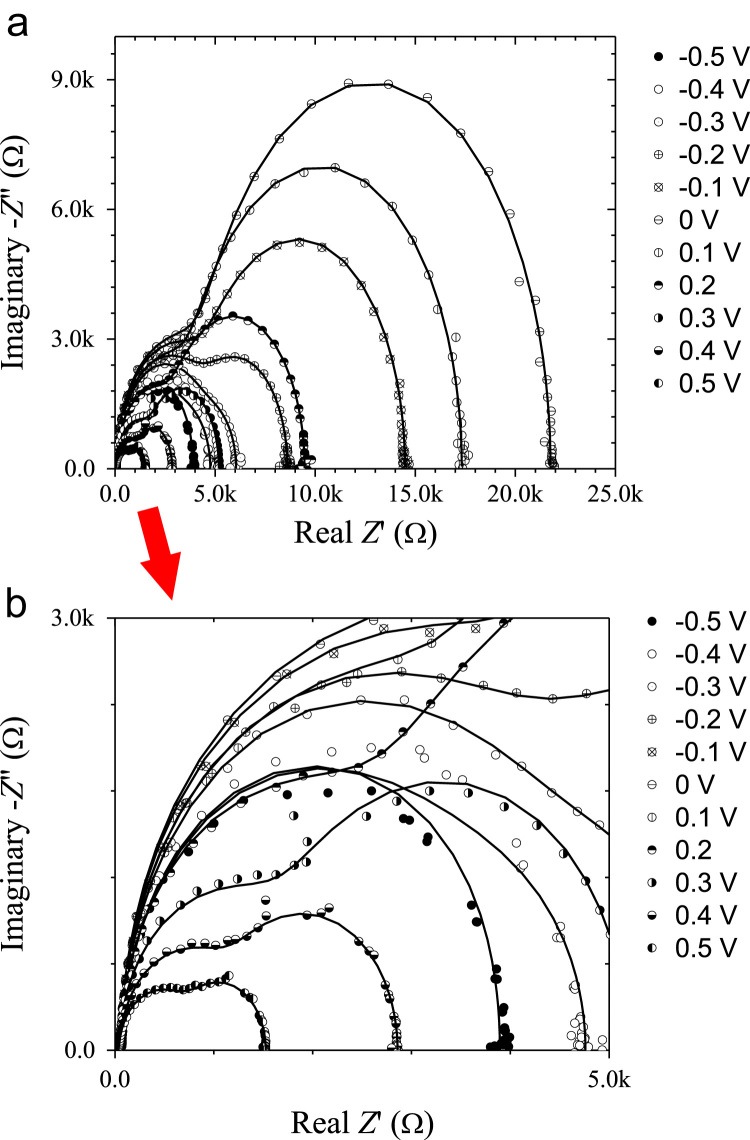
Nyquist (Cole–cole) plots as a function of bias of ITO/AZO/Ge device for the real impedance range of (a) 0–25 kΩ (b) for 0–5 kΩ.

**Fig. 4:**
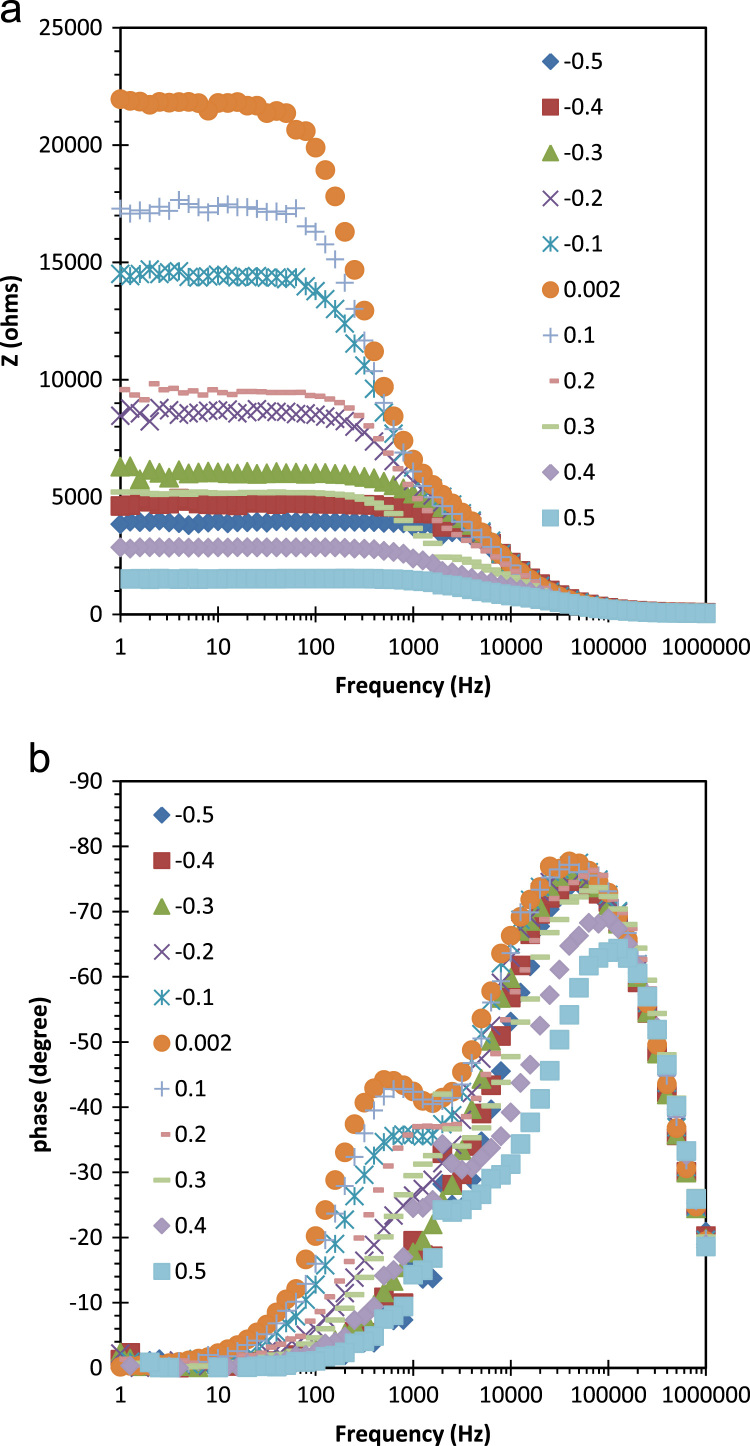
(a) Bode magnitude, and (b) Bode phase plots as a function of bias.

**Fig. 5:**
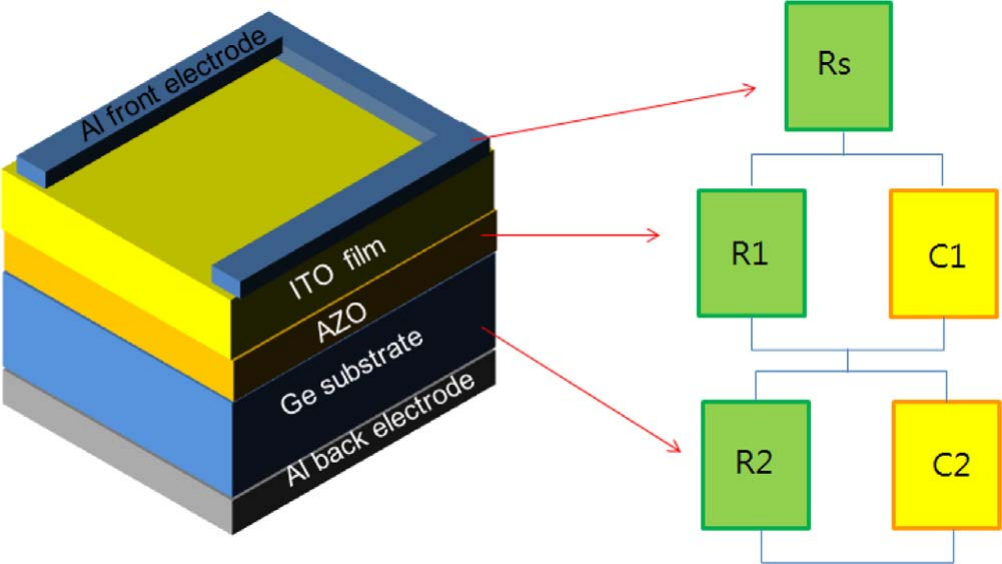
Equivalent RC circuit model for obtaining best fit out of impedance spectra of ITO/AZO/Ge photodetector.

Fig. 1 shows free carrier concentrations in Ge wafer as a function of built in potential. These data are estimated using the Mott–Schottky characteristics at various frequency. This results revealed the frequency dependent of the majority carrier concentration as well as built in potential. Here negative sign of carrier concentration denotes hole as majority type. Cole–Cole plots of ITO/AZO/Ge device by modulation in the applied bias. The series and parallel resistance, and series and parallel capacitance and there frequency dependent data are presented in Fig. 2. Bias dependent Nyquist plots are shown Fig. 3. In these data, imaginary impedance (*Z*′′) and real impedance (*Z*′) are shown on *y*-axis and *x*-axis, respectively. There relation to the frequency can be found in Fig. 4, which denotes as Bode-magnitude (Fig. 4a) and Bode-phase (Fig. 4b) plot. The data shown in Fig. 3, Fig. 4, were applied to the equivalent R-C circuit as presented in the Fig. 5. The R-C circuit was modeled by the space charge region (SCR) and resistance elements. Table 1 presents the equivalent resistance and capacitance values of the R-C circuit.

**Table 1:** The values R1C1 and R1C2 circuits along with series resistance for the voltage range of −0.5 to 0.5 V.

**Voltage (V)**	***Rs*****(ohm)**	***R*****1 (ohm)**	***C1*****(nF)**	***R2*****(ohm)**	***C2*****(nF)**
−0.5	70	741	115	3142	6.3
−0.4	71	1386	73.5	3302	6.5
−0.3	69	1593	130	4386	6.7
−0.2	62	4232	74	4320	7.1
−0.1	63	10,061	52	4363	7.5
0.0	62	17,486	42	4209	7.9
0.1	56	13,626	43	3640	8
0.2	55	6701	50	2713	8.2
0.3	45	3481	53	1661	8.2
0.4	48	1708	68	1102	8.5
0.5	42	809	80	673	8.8

## 2 Experimental design, materials and methods

### 2.1 Free carrier concentration and built in potential of ITO/AZO/Ge device

Mott-Schottky analyses of the ITO/AZO/Ge device [1] were performed in the frequency range of 1–20 kHz at the forward or reverse bias condition at room temperature. The estimated free carrier concentration of the device is shown in Fig. 1 as a function of built in potential. The negative value of free carrier concentration denotes the hole majority carriers [2].

### 2.2 Series and parallel measurements of ITO/AZO/Ge device

The measured parallel capacitance (*C*_p_) and resistance (*R*_p_) of the ITO/AZO/Ge device is shown Fig. 2a. The measured series capacitance (*C*_S_) and resistance (*R*_S_) of the ITO/AZO/Ge device is shown Fig. 2b.

### 2.3 Cole–Cole plot of ITO/AZO/Ge device

Impedance spectra of the ITO/AZO/Ge device were analyzed in the frequency range of 1 Hz to 20 kHz under forward and reverse dc biases (from −0.5 V to +0.5 V) at room temperature. The Cole–Cole plots of the prepared device were shown in Fig. 3. The real impedance range of 0–5 kΩ (for the forward bias region) has been focused to the corresponding imaginary part of impedance in Fig. 3. In order to show the frequency effect, the Bode-magnitude and Bode-phase plots were shown in Fig. 4a and b, respectively.

### 2.4 Equivalent R-C circuit analyses

The total impedance of the device is referred from the radius of the semicircles. In the ideal diodes, the impedance increases in the forward bias and decreases under reverse bias. In contradict to the ideal one; the present device showed the larger impedance at zero bias and it is gradually decreased under both forward and reverse biases. However, the minimum impedances were experienced by the device under the forward bias voltages (0.5, 0.4, 0.3 and 0.2 V). This indicates that the impedance of the device depends on the applied DC bias voltage. The impedance spectra over the DC bias range of −0.5 to 0.5 V are almost semi-circular in shape which implies that the Schottky junction can be represented by an equivalent circuit model consisting of resistances and capacitances (R–C).

The simplest ideal Schottky junction is typically equivalent to a parallel connected R-C circuit with a series resistance. The R-C circuit represents the SCR and series resistance represents the Ohmic contacts (or) bulk resistance of the device. For other commercial devices, more than one parallel connected RC circuit may be connected in series to obtain the best fit out of the impedance spectra. The number of RC circuits used in the equivalent circuit model mainly depends on the structure of the prepared device. Besides, if the semi-circles in the impedance spectra are perfect in shape, then it is understood that there is only one parallel connected R–C circuit in the equivalent model. However, in the present case, the impedance spectra do not have perfect semi-circular shape, whereas, each impedance spectrum consists of one large and one small semicircle. This made our approximation quite simple. That is, the equivalent RC circuit model of the prepared ITO/AZO/Ge device consists of two parallel connected RC networks with a series resistance as shown in Fig. 2.

The R1C1 circuit is assigned to the SCR located at the AZO/Ge interface and the R2C2 is assigned to the interface between Ge and back Al electrode. The values of R1, C1, R2, C2 and series resistance corresponding to the applied bias voltage is given in Table 1. In the first RC circuit, the resistance (R1) at SCR decreases while C1 increases under both forward and reverse bias. However, both the C2 and R2 are decreased under reverse bias in the second RC circuit. This infers that the width of SCR expands under reverse bias so that it reduces the value of C2. Yet, the carriers are tunneled through the barrier due to the reducing R2 values in the reverse bias. This contributes to the reverse saturation current. This analysis highlights the fact that the charge collection and separation processes take place only at the interface of AZO and Ge due to the increasing capacitance value of C1.
